# Isolation of Endophytic Phosphate-Solubilizing Bacteria from Chinese *Cymbidium* (*Cymbidium* spp.) Orchid Roots

**DOI:** 10.3390/microorganisms13102229

**Published:** 2025-09-23

**Authors:** Yanmei Sun, Jianpeng Jin, Xiting Wang, Wei Zhu, Jie Gao, Jie Li, Qi Xie, Yonglu Wei, Chuqiao Lu, Genfa Zhu, Fengxi Yang

**Affiliations:** 1 Guangdong Key Laboratory of Ornamental Plant Germplasm Innovation and Utilization, Environmental Horticulture Research Institute, Guangdong Academy of Agricultural Sciences, Guangzhou 510640, China; 2 College of Horticulture and Forestry, Huazhong Agricultural University, Wuhan 430070, China

**Keywords:** endophytic phosphate-solubilizing bacteria, biofertilizer potential, plant growth-promoting bacteria, Chinese *Cymbidium*

## Abstract

This study aims to identify and evaluate the phosphate-solubilizing ability of endophytic bacteria isolated from roots of Chinese *Cymbidium* and to assess their impact on phosphorus uptake and plant growth. Thirty strains of endophytic bacteria were isolated from six orchid varieties. Molecular identification based on 16S rRNA gene sequencing revealed that the most frequently isolated strains belonged to the genera *Pseudomonas* and *Burkholderia*. Among them, 10 bacterial strains exhibited the capacity to solubilize inorganic and organic phosphorus. Two strains, designated X1 (*Paraburkholderia* sp. Beta-32) and X13 (*Rhizobium freirei* PRF81 (X13), were identified as the most effective phosphate-solubilizing bacteria (PSB). Gluconic acid was the dominant organic acid secreted, driving inorganic phosphorus solubilization, while alkaline phosphatase activities facilitated organic phosphorus mineralization. Inoculation with phosphate-solubilizing bacteria (PSB) resulted in increased plant growth and phosphorus content in both leaves and roots as compared to the control plants. PSB treatments also increased available phosphorus content in soil, reduced total phosphorus content, and increased exopolysaccharide and alkaline phosphatase activities. Real-time q-PCR analysis showed that PSB inoculation significantly upregulated the expression of phosphorus transport-related genes, including *PDR2*, *PHF1*, *PHR1*, *PHT1;9*, and *PHT4;4*, thereby enhancing phosphorus absorption. Moreover, strains X1 and X13 not only exhibited strong phosphate-solubilizing capacity but also demonstrated stable colonization in both roots and root rhizosphere soil of orchids over extended periods. In conclusion, the endophytic PSB identified with phosphate-solubilizing abilities increased phosphorus availability and its uptake in Chinese *Cymbidium*, thereby promoting plant growth and development. This is the first attempt to characterize endophytic PSB from roots of Chinese *Cymbidium* orchids. These findings provide a basis for selection of PSB that are efficient in P uptake for application in microbial fertilizers for orchid cultivation.

## 1. Introduction

Chinese *Cymbidium* (*Cymbidium* spp.), also known as Chinese orchid, has an extensive cultivation history in China. It ranks among the top commercial flowers in the country due to its distinctive aroma and cultural significance [1]. Given the multifaceted importance of Chinese *Cymbidium*, enhancing its growth, flower yield, and quality—including the number, size, and longevity of blooms—could substantially benefit growers financially. Floriculture production often relies heavily on synthetic fertilizers containing nitrogen (N), phosphorus (P), and potassium (K). Among these nutrients, phosphorus plays a crucial role as a precursor of sugar phosphates during respiration and photosynthesis and as a precursor of phospholipids that make up plant membranes [2,3,4,5,6]. Phosphorus is also vital for cell division, development of meristematic tissues, and stimulation of bud and bulb formation [7]. The primary goal of phosphorus fertilization in ornamental plants is to enhance growth and cultivate healthy, robust development. However, the high cost of phosphorus fertilizers limits their widespread use [8]. Moreover, prolonged application of these fertilizers can degrade soil structure and ultimately have a negative impact on flower growth and yield [8,9]. Consequently, there is a need to identify cost-effective and environmentally friendly alternatives to the use of chemicals in floriculture practices. This has prompted us to focus on investigating the use of phosphate-solubilizing bacteria (PSB) to promote growth of Chinese *Cymbidium*.

PSB has the potential to convert insoluble phosphates available to plants through solubilization and mineralization, increasing crop yields, while maintaining environmental sustainability [10]. Genera such as *Pseudomonas*, *Burkholderia*, *Bacillus*, *Rhizobium*, *Sphingobacterium*, and *Stenotrophomonas* have been shown to exhibit phosphate-solubilizing ability along with plant growth-promoting traits, rendering them promising candidates for biofertilizer development [8,10,11,12]. PSB converts both insoluble inorganic and organic phosphorus into soluble active phosphorus, a process associated with the secretion of various organic acids, exopolysaccharide, and extracellular enzymes such as phosphatases [11,12,13,14,15,16,17,18,19]. The secretion of various organic acids by PSB is considered the primary mechanism of phosphorous solubilization, with gluconic acid being the main component during this process [11]. PSB affects the expression of genes related to phosphorus starvation responses and high-affinity phosphorus transporters in plants. Several genes, including *PDR*, *PHF*, *PHO*, *PHT*, and *SPXs*, are involved in the absorption, transport, and redistribution of inorganic phosphorus, improving host nutrient absorption and promoting growth and development [20,21,22].

The use of PSB has been shown to increase plant phosphorus uptake and yield in numerous studies across a variety of crops, including rice, wheat, and maize [23,24,25,26]. The majority of these PSB originate from the soil environment surrounding crop roots, known as the rhizosphere. Endophytic bacteria share a more intimate relationship with plants than soil bacteria. As endophytic PSB are isolated from root tissues, they may possess a superior ability to colonize the host plant and potentially facilitate the mobilization of internal phosphorus pools in addition to solubilizing phosphorus in the soil. Orchidaceous plants, including the Chinese *Cymbidium*, are a special group of plants that have a symbiotic relationship with endophytes [27,28]. Because the seeds of orchids are small, the embryos are not fully differentiated, have little endosperm, and therefore rely on endophytic microorganisms to provide nutrients to promote germination and subsequent protocorm growth [27,28,29,30].

The unique habitat of endophytic PSB in Chinese *Cymbidium* is an important but underdeveloped phosphate-soluble microbial resource. The endophytic bacteria with phosphate-solubilizing functions in Chinese *Cymbidium* have not been studied systematically. The aim of this research work is to screen for endophytic bacteria that can dissolve inorganic phosphorus and mineralize organic phosphorous from roots of Chinese *Cymbidium*. These findings provide a comparison of efficient PSB in P uptake as microbial fertilizers which can be used in production of Chinese *Cymbidium*.

## 2. Materials and Methods

### 2.1. Sample Collection and Treatment

Roots from six orchid plantlets of different hybrids were collected from the orchid germplasm resources of the Environmental Horticulture Research Institute, Guangdong Academy of Agricultural Sciences (23°8′ N113°20′ E) (Table S1), in February 2024. Three root samples were obtained from each plantlet in sterile plastic bags, washed with sterilized water, and dried at room temperature. Each sample was surface-sterilized with 75% ethanol for 2 min, with 10% (*v*/*v*) sodium hypochlorite solution for 10 min, and then was washed with sterilized water three times [31]. The washed root tissues were gently placed on nutrient agar (NA) (containing (L^−1^): peptone, 10 g; beef extract, 3 g; NaCl, 5 g; 20 g of agar; 1000 mL of distilled water (pH 7.0)) solid medium and incubated at 30 °C to test for effectiveness of surface sterilization. If no colonies grew on the surface of the NA solid medium within 3 d, it indicated that the surface sterilization was successful. Simultaneously, the roots were homogenized in a sterile grinder and suspended in 1 mL of sterile water, which served as the suspension medium, followed by thorough vortexing to form a uniform suspension. Serial dilutions were then prepared to obtain 10^−1^, 10^−2^, 10^−3^, 10^−4^, and 10^−5^ gradient dilutions. A 50 μL aliquot from each dilution was aseptically spread onto NA solid medium and incubated at 30 °C for 3 d. Colonies grown on the NA solid medium were purified and stored at 4 °C.

### 2.2. DNA Extraction and PCR Amplification

The extraction of endophytic bacterial DNA and sequencing were performed according to a previously described method [32]. The 16S ribosomal RNA gene of each isolate was amplified using the universal primers 27F (5′-AGAGTTTGATCCTGGCTCAG-3′) and 1492R (5′-CTACGGCTACCTTGTTACGA-3′) [33] under the following PCR conditions: 94 °C for 5 min, followed by 35 cycles of 94 °C for 30 s, 50 °C for 30 s, 72 °C for 1 min, and a final elongation step at 72 °C for 10 min. Initial isolation yielded over 70 bacterial strains from Chinese *Cymbidium* roots. All isolates were characterized by amplifying 16S rDNA using universal bacterial primers. PCR products of expected size were electrophoresed, excised, purified, and sequenced. The resulting sequences were analyzed using BLAST (http://www.ncbi.nlm.nih.gov/, accessed on 23 May 2024), against the NCBI database [34]. Redundant strains were eliminated based on sequence comparisons, yielding 30 phylogenetically distinct endophytes. These selected strains were preserved in 50% glycerol at −80 °C and are listed in Supplementary Table S1. A phylogenetic tree was constructed using the maximum likelihood method with the MEGA 7.0 software [35].

### 2.3. Selection of PSB Strains in Solid and Liquid Media

To measure the phosphate-solubilizing ability of 30 strains of PSB, they were inoculated on inorganic phosphorus (IP) and organic phosphorus (OP) medium. IP media contained (L^−1^), which was composed of the following: C_6_H_12_O_6_, 10.0 g; (NH_4_)_2_SO_4_ 0.5 g; FeSO_4_·7H_2_O, 0.03 g; yeast extract, 0.5 g; NaCl, 0.3 g; KCl, 0.3 g; MnSO_4_·4H_2_O, 0.03 g; MgSO_4_·7H_2_O, 0.3 g; Ca_3_(PO_4_)_2_, 5.0 g; 20 g of agar; 1000 mL of distilled water (pH 7.0). OP was similar to OP medium except that 2 g lecithin and 5 g CaCO_3_ were added instead of calcium phosphate. The strains that were able to form halo zones on both IP and OP solid medium were considered positive strains. The accession numbers of these positive strains in the NCBI database are listed in Supplementary Table S2. Initially, positive strains were grown on IP and OP solid medium plates and incubated at 30 °C for 5 d. Measurement of the phosphate solubilization halo zone diameter (D) and colony diameter (d), followed by calculation of the ratio between halo zone diameter and colony diameter (D/d) was carried out [36]. Subsequently, the phosphate-solubilizing ability of the strains was evaluated using a liquid media method [37]. First, each bacterial strain was cultured to a standardized concentration of OD_600_ = 0.8–1.0; then, 200 uL of each standardized culture was inoculated into 100 mL of IP or OP liquid medium and incubated at 30 °C for 5 d on a rotary shaker at 180 rpm. Uninoculated media (with 200 uL of sterile water added) served as negative controls (initial pH, 7.0; final pH, 7.0). Experiments were repeated three times for each strain. The IP and OP liquid medium were centrifuged at 10,000 rpm for 10 min. The IP medium was analyzed for pH, soluble phosphorus, organic acids, and exopolysaccharides, while the OP medium was analyzed for soluble phosphorus and phosphatase activities.

### 2.4. Measurement of Plant Growth Parameters

To study the effects of selected endophytic PSB on the growth and development of orchids, plant experiments were carried out in the greenhouse. After washing and centrifugation steps to remove the culture medium, the bacterial pellet was resuspended in sterile water to an OD_600_ of 0.8–1.0. This bacterial suspension was then diluted 10-fold with sterile water to achieve the working concentration. In the subsequent pot experiments, we employed a completely randomized design to evaluate ten different PSB strains, with sterile water serving as the negative control treatment. The tissue culture plantlets were dipped and incubated in endophytic PSB for 12 h and then planted in the pot. Each pot contained 250 g of pine bark (pH 6.5) and was planted with one orchid plantlet. There were 15 pots for each treatment, and 5 mL of the bacterial suspension (working concentration) was added to the pine bark. To meet the nutritional requirements of the plantlets, they were irrigated with modified 1/2 phosphorus-deficient Hoagland’s nutrient solution. Additionally, 2.5 g·L^−1^ tricalcium phosphate and 1 g·L^−1^ lecithin were supplemented per liter of phosphorus-deficient Hoagland’s nutrient solution (KNO_3_, 607 g·L^−1^; MgSO_4_, 241 mg·L^−1^; NH4NO_3_, 40 mg·L^−1^; FeNaEDTA, 36.7 m·L^−1^; KI, 0.83 mg·L^−1^; H_3_BO_3_, 6.2 mg·L^−1^; MnSO_4_·H_2_O, 22.3 mg·L^−1^; ZnSO_4_·7H_2_O, 8.66 mg·L^−1^; Na_2_MOO_4_·10H_2_O, 0.25 mg·L^−1^; CuSO_4_·7H_2_O, 0.025 mg·L^−1^; CoCl_2_, 0.025 mg·L^−1^; Ca(NO_3_)_2_, 945 mg·L^−1^; 20 g of agar; 1000 mL of distilled water (pH 6.0) as a phosphorus source substitute for potassium dihydrogen phosphate). Watering (250 mL) was carried out once every two weeks. At 30 d of co-cultivation, the orchid plantlets in the treatment groups were harvested to measure leaf and root lengths. Leaf length was measured from the lamina base to the apex in fully expanded leaves, and root length was calculated as primary root length from the hypocotyl junction to the distal tip. Subsequently, part of the leaves and roots were washed several times with deionized water, and the dry weight was recorded after drying at 80 °C for 7 d. Rhizosphere soils were sampled at 30 d after treatment for measurements of total phosphorus, available phosphorus, and pH. Roots were sampled at 30 d after treatment for measurements of phosphorus content, exopolysaccharides, and phosphatase activities.

### 2.5. Measurements of Soluble Phosphorus in Liquid Medium and Total Available Phosphorus in Rhizosphere Soils

The analysis of soluble phosphorus concentrations in IP and OP liquid medium was performed using the previous method [37]. After centrifugation of the bacterial strain culture, 1 mL of supernatant was transferred to a test tube. Then, 20 μL of a 10 mM 2,4-dinitrophenol indicator (Product No. D835613, Macklin Biochemical, Shanghai, China; CAS 51-28-5) was added. Subsequently, 5 mL of molybdate/antimony reagent was accurately added, the mixture was shaken well, and distilled water was added to bring the volume up to 7 mL. Following this, the test tubes were incubated for 30 min at room temperature. The appearance and intensity of the blue color indicated the total concentration of phosphorus, and the absorbance was measured at 700 nm. The phosphorous concentrations in the test plants were determined using the molybdenum blue method [38]. Total phosphorus (TP) and available phosphorus (AP) in rhizosphere soil samples were determined by sulfuric acid–perchloric acid decoction and NaHCO_3_ extraction was carried out using the molybdenum antimony colorimetric method [39]. The pH variations were measured using a pH meter (Mettler-Toledo GmbH, Greifensee, Switzerland).

### 2.6. Measurements of Exopolysaccharides

Analyses of exopolysaccharides in IP liquid medium and rhizospheric soils were performed using Gauri’s method [40]. Exopolysaccharides (EPSs) were precipitated in the supernatant by adding two volumes of isopropanol and centrifuged at 10,000 rpm for 10 min at 4 °C. After removing the supernatant, the sediment was dissolved in 2 mL of distilled water, and 1 mL of 6% phenol solution was added, followed by vigorous shaking and rapid addition of 5 mL of concentrated sulfuric acid. The mixtures were immediately shaken again and left to stand at room temperature for 30 min, and the absorbance was measured at 490 nm.

### 2.7. Analysis of Organic Acid Production in Liquid Cultures by HPLC

The production of organic acids in bacterial cultures carried out in IP liquid medium was performed with the methodology used by Fiori and his co-workers [41]. After centrifugation of the bacterial strain culture, 1 mL of supernatant was transferred to a test tube. Then, 500 μL of 30% methanol aqueous solution containing 0.1% formic acid was added; the mixture was shaken, mixed vigorously for 60 s, and then centrifuged at 12,000 rpm at 4 °C for 10 min. The sample was filtered through 0.22 μm membranes, and the filtrate was added to the LC-MS bottle. An amount of 5 uL was injected into a high-performance liquid chromatography (HPLC) system (Agilent 1100 series; Agilent Technologies, Santa Clara, CA, USA) equipped with an ACQUITY UPLC^®^ BEH C18 column (2.1 × 100 mm, 1.7 μm, Waters Corporation, Milford, MA, USA). The solution of 0.1% formic acid in water (A) and methanol (B) was used as the mobile phase at 0.4 mL·min^−1^ flow rate. The organic acid secretion of bacterial strains was identified through a comparison of the retention times, and the quantification was performed by the comparison of the peak areas using external standards.

### 2.8. Measurements of Alkaline Phosphatase Activities

Alkaline phosphatase activities in both OP liquid medium and rhizosphere soils were analyzed according to Qvirist et al. [42]. After 5 d incubation in OP liquid medium, 10 mL of bacterial culture was transferred to a 15 mL conical centrifuge tube. For soil samples, 2 g was homogenized with 10 mL of sterile distilled water in a 15 mL tube and incubated statically for 1 h. Both sample types were then centrifuged at 10,000 rpm for 10 min. Supernatant of extract (0.1 mL) was mixed in 2 mL of modified universal buffer (pH = 11) and 0.5 mL of p-nitrophenyl phosphate (*p*-NP) substrate solution (50 mM). The change in the alkaline phosphatase solution due to p-nitrophenol (*p*-NP) production was measured at 400 nm, and the amount of *p*-NP was calculated from a *p*-NP calibration curve. Solution without soil served as control. One unit of phosphomonoesterase activities was defined as the amount of enzyme required to liberate 1 mM of *p*-NP (product) from 1 kg of dried soil/h at 37 °C. Protein concentration was determined using Coomassie Brilliant Blue G-250 solution as previously described [43].

### 2.9. Colonization Dynamics of PSB in Orchid Roots and Rhizosphere Soil

The rifampicin-resistant marker method [44] was employed to screen for the marker strains grown on NA solid medium containing 300 μg·mL^−1^ rifampicin, with colony morphology consistent with that of the original strains. Following the methods of previous studies for strain isolation and recovery, the target strains were isolated from groups of inoculated and non-inoculated orchids after 30 d. A sample of 1 g each of orchid roots and rhizosphere soil was weighed and added to 9 mL of sterile water for dilution. An amount of 100 μL of the soil was diluted at 10^−2^, 10^−3^, and 10^−4^ and evenly spread on NA agar plates containing 300 μg·mL^−1^ rifampicin. After incubation at 30 °C for 3 d, the number of colonies on each plate was counted and converted into number of colony-forming units per gram of plant roots and dry soil (cfu·g^−1^). Each treatment was replicated three times.

### 2.10. Quantitative RT-PCR Analysis and Statistical Analysis

Total RNA was extracted using RNAprep pure Plant Kit (Tiangen Inc., Beijing, China). cDNA was synthesized using HiScript III RT SuperMix for qPCR Kit (Vazyme, Nanjing, China) according to the manufacturer’s protocol. Quantitative RT-PCR was conducted on the Thermal Cycler Dice^TM^ Real-Time System (Takara, Otsu, Japan) using the diluted cDNA as templates. The primers were designed using Primer5 software. The β-actin (Mol013347) was used as an internal reference gene to normalize the amount of template in *Cymbidium sinense* as described, and Actin was also used as control [45]. All primers and their sequences are listed in Supplemental Table S2. Relative expression was calculated based on the 2^−ΔΔCt^ method [46]. Each experiment contained two biological replicates and three technical replicates.

### 2.11. Statistical Analysis

The physiological measurements were repeated three times from different samples. A full random design model on the SPSS software (v28.0.1.1; SPSS Inc., Chicago, IL, USA) was used to analyze the variance of data. Differences among means of samples were evaluated using Duncan’s test at *p* < 0.05 level.

## 3. Results

### 3.1. Bacterial Identification

Thirty strains of endophytic bacteria were isolated from roots of six Chinese orchids. The nucleotide sequences of the 16S rRNA genes of the 30 strains were amplified by PCR and sequenced. These sequences were compared by BLAST with sequences in the NCBI database, and the results are shown in Figure S1. These results showed that the 30 strains of bacteria belonged to six genera, including 11 strains of *Pseudomonas*, 8 strains of *Burkholderia*, 7 strains of *Bacillus*, 1 strain of *Sphingobacterium*, 1 strain of *Rhizobium*, and 1 strain of *Stenotrophomonas maltophilia*. Two additional isolates were only broadly classified as *Bacterium* sp. (Figure S1).

The IP and OP were chosen as selective culture mediums to screen for phosphate-solubilizing ability of 30 strains of endophytic bacteria. Out of these, 17 strains of endophytic bacteria could form clear phosphate-solubilizing halos on IP solid medium, and 10 strains of endophytic bacteria could form clear phosphate-solubilizing halos on OP solid medium. Therefore, 10 strains of endophytic bacteria were identified as being capable of decomposing both inorganic phosphorus and mineralizing organic phosphorus. These strains included *Paraburkholderia* sp. strain Beta-32 (X1), *Pseudomonas* sp. strain SWUSTb-72 (X2), *Burkholderia ambifaria* strain AU0212 (X9), *Rhizobium freirei* PRF81 (X13), *Burkholderia fungorum* (X16), *Pseudomonas brenneri* strain EH-G3 (X20), *Paraburkholderia phytofirmans* PsJN (X21), *Bacillus thuringiensis* strain EI-17 (X27), *Bacillus wiedmannii* strain ASS-1 (X29), and *Burkholderia tropica* strain CACua-41 (X30) (Table S1).

### 3.2. Evaluation of Phosphate-Solubilizing Ability

#### 3.2.1. Ability to Dissolve Inorganic Phosphorus

**Figure 1:**
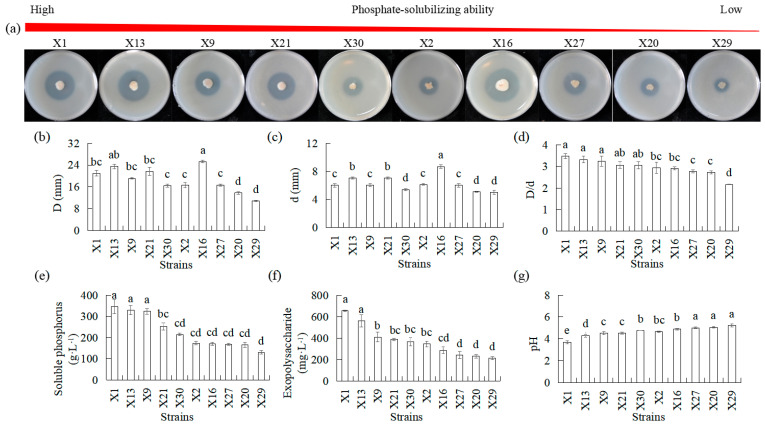
Comparison of inorganic phosphate-solubilizing capacities of different endophytic PSB in Chinese *Cymbidium*. (**a**) PSB phosphate-solubilizing ability was visualized on IP solid medium through halo zone formation, followed by measurements of (**b**) halo zone diameter D and (**c**) colony diameter d. (**d**) The ratio between halo zone diameter and colony diameter D/d was calculated. For IP liquid medium, (**e**) measurements of soluble phosphorus, (**f**) exopolysaccharide, and (**g**) pH. Means of three replicates and standard errors are presented; the same letter above the column indicates no significant difference at *p* < 0.05.

Ten isolates representing endophytic PSB were analyzed over a period of 5 d in terms of their phosphate-solubilized halo zone diameters, colony diameters, D/d, pH, exopolysaccharide, and soluble phosphorus using Ca_3_(PO_4_)_2_ as the insoluble phosphorous sourced (IP solid medium) (Figure 1). Phosphate-solubilized halo diameters ranging from 13.68 to 25.2 mm and colony diameters ranging from 5.1 to 8.7 mm were observed 5 d after inoculation, which resulted in D/d values of 1.9 to 3.48. The differences in phosphate-solubilizing halos and colony diameters among the 10 endophytic PSB were significant. Strains X1, X13, X9, X21, and X30 had a higher D/d than X27, X20, and X29, and all PSB exhibited more than 1.5 in D/d (Figure 1a–c). There were significant differences in pH, exopolysaccharide, and soluble phosphorus in the 10 endophytic PSB. Exopolysaccharide and soluble phosphorus were increased after inoculation. Higher levels of increase were observed in X1, X13, and X9 than in X2, X6, X27, X20, and X29 (Figure 1e,f). The pH decreased after inoculation. Lower levels were observed in X1, X13, and X9 than in X30, X2, and X6, which in turn were lower than those in X27, X20, and X29 (Figure 1d).

Since the pH values of all strains decreased (Figure 1), we then measured the content of organic acids. Several organic acids were identified in the culture supernatants of endophytic PSB, most of which were glucuronic acid, lactic acid, citric acid, tartaric acid, and pantothenic acid in descending amounts (Table 1). The types of organic acids secreted by different bacteria were different, and the ability to secrete the same acid varies, but the total organic acid secretion capacities were X1 > X13 > X9 > X21 > X30 > X2 > X16 > X27 > X20 > X29. Higher levels of fumaric acid, succinic acid, pyroglutamic acid, glutaric acid, malic acid, 5-hydroxymethyl-2-furoic acid, tartaric acid, pyridoxine, citric acid, and glucuronic acid were observed in X1 as compared to X27, X20, and X29. Higher levels of lactic acid, fumaric acid, succinic acid, malic acid, phenylpyruvic acid, phenyllactic acid, glucuronic acid, and pantothenic acid were observed in X13 compared to X27, X20, and X29. Among them, fumaric acid, succinic acid, malic acid, and glucuronic acid were higher in X1 and X13 than in X27, X20, and X29.

#### 3.2.2. Ability to Mineralize Organic Phosphorus

**Figure 2:**
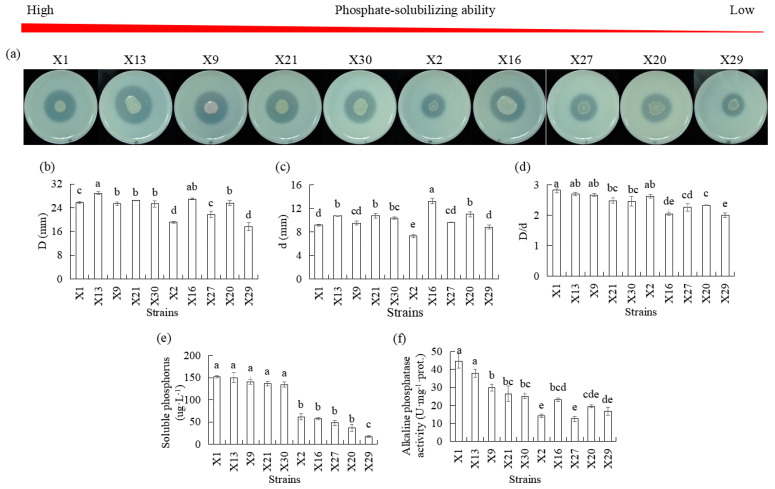
Comparison of organic phosphate-solubilizing capacities of different endophytic PSB in Chinese *Cymbidium.* (**a**) PSB phosphate-solubilizing ability was visualized on OP solid medium through halo zone formation, followed by measurements of (**b**) halo zone diameter D and (**c**) colony diameter d. (**d**) The ratio between halo zone diameter and colony diameter D/d was calculated. For OP liquid medium, measurements of (**e**) soluble phosphorus and (**f**) alkaline phosphatase activities. Means of three replicates and standard errors are presented; the same letter above the column indicates no significant difference at *p* < 0.05.

Ten isolates representing endophytic PSB were studied over a period of 5 d in terms of their phosphate-solubilized halo zone diameters, colony diameters, D/d, alkaline phosphatase activities, and soluble phosphorus concentration. An amount of 2 g of lecithin and 5 g of CaCO_3_ was added instead of calcium phosphate (OP solid medium) as the organic phosphorous sourced (Figure 2). Phosphate-solubilized halo diameters ranging from 17.6 to 28.9 mm and colony diameters ranging from 7.3 to 11.0 mm were observed after inoculation, which resulted in D/d values of 2.0 to 2.82. The differences in phosphate-solubilizing halos and colony diameters among the 10 strains were significant. Strains X1, X13, and X9 had a higher D/d than X27, X20, and X29, and all PSB exhibited more than 2.0 in D/d (Figure 2b–d).

We determined the content of alkaline phosphatase activities, and higher levels were observed in X1, X13, and X9 than in X2, X6, X27, X20, and X29, and the alkaline phosphatase activities secreted by X2 and X27 were the lowest (Figure 2f). There were significant differences in soluble phosphorous concentration levels among the 10 endophytic PSB strains. The soluble phosphorous concentration increased after inoculation, with higher levels observed in the X1, X13, and X9 strains as compared to the X2, X6, X27, X20, and X29 strains (Figure 2e).

### 3.3. Effects of Endophytic PSB Inoculation on P Uptake and Orchid Growth

**Figure 3:**
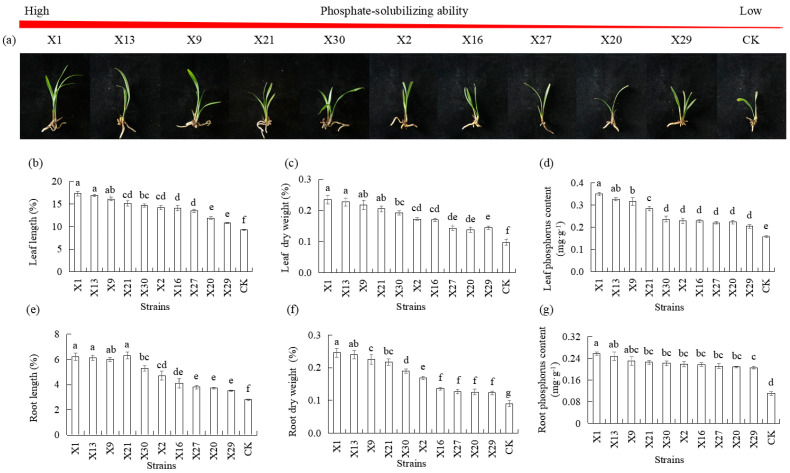
Growth response of Chinese *Cymbidium* seedlings inoculated with different endophytic PSB. (**a**) Morphological characteristics photographs of Chinese *Cymbidium* seedlings after 30 d of PSB inoculation, followed by measurements of (**b**) leaf length, (**c**) leaf dry weight, (**d**) leaf phosphorus content, (**e**) root length, (**f**) root dry weight, and (**g**) root phosphorus content. CK represents the non-inoculated treatment (control group). Means of three replicates and standard errors are presented; the same letter above the column indicates no significant difference at *p* < 0.05.

Plant growth was measured to evaluate the phosphate-solubilizing ability of PSB after plantlets were inoculated with PSB for 30 d. Plants inoculated with endophytic PSB showed significantly higher growth in leaf length, root length, leaf dry weight, and root dry weight compared to the uninoculated endophytic PSB (CK) (Figure 3a). Plants inoculated with the X1, X13, and X9 strains had higher growth than plants inoculated with the X20 and X29 strains (Figure 3b,c,e,f). The increase in phosphorus content was observed in the treatment of endophytic PSB, which resulted in an increase in phosphorus content in leaf and root compared to plants not inoculated with endophytic PSB (CK). Plants inoculated with the X1, X13, and X9 strains had higher phosphorus content in leaves and in roots compared to plants inoculated with the X20 and X29 strains (Figure 3d,g).

### 3.4. Effects of Endophytic PSB Inoculation on the Properties and Phosphorus Availability in the Rhizosphere Soil of Orchids

**Figure 4:**
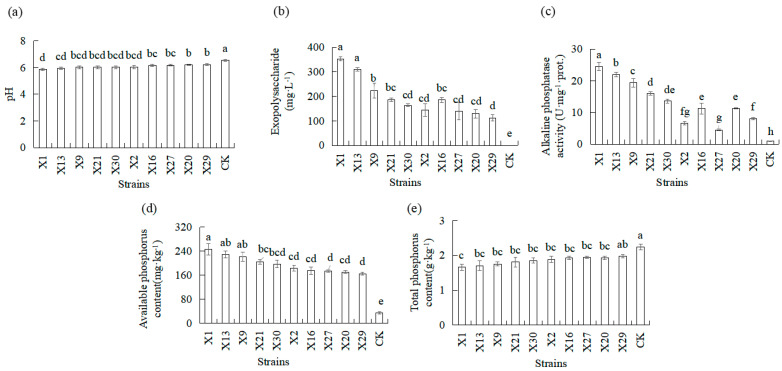
Physical and chemical properties response of Chinese *Cymbidium* rhizosphere soil inoculated with different endophytic PSB. Rhizosphere soil properties were measured in terms (**a**) pH, (**b**) exopolysaccharide, (**c**) alkaline phosphatase activities, (**d**) available phosphorus, and (**e**) total phosphorus. CK represents the non-inoculated treatment (control group). Means of three replicates and standard errors are presented; the same letter above the column indicates no significant difference at *p* < 0.05.

The available phosphorus content, total phosphorus content, pH, exopolysaccharide, and alkaline phosphatase activities in rhizosphere soils were measured to evaluate phosphate-solubilizing ability in plants inoculated and not inoculated with PSB (Figure 4). Plants’ inoculation with PSB resulted in increased available phosphorus content, as well as in decreased total phosphorus content in rhizosphere soils, compared to plants not inoculated with PSB (CK). PSB treatments of orchids produced an increase of 381–619% in available phosphorus content, and total P content in rhizosphere soils decreased 11.8–25.8% compared to uninoculated plants (CK). The X1 and X13 strains demonstrated the maximum increase in available phosphorus content in rhizosphere soils, and the X1 and X13 strains demonstrated the maximum decrease in total phosphorus content in soil (Figure 4d,e). Plants’ inoculation with PSB resulted in decreased pH in soil compared to plants not inoculated with PSB (CK). The maximum decrease in pH in soil was recorded in plants inoculated with the X1 and X13 strains compared to the X27, X20, and X29 strains (Figure 4a). Inoculation with PSB significantly affected the exopolysaccharide and alkaline phosphatase activities of orchids compared to uninoculated orchids (CK). Higher levels were observed in the X1, X13, and X9 strains compared to the X2, X6, X27, X20, and X29 strains (Figure 4b,c).

### 3.5. Gene Transcript Levels Involved in Phosphorus Transport in Chinese Cymbidium Inoculated with Different PSB Strains

**Figure 5:**
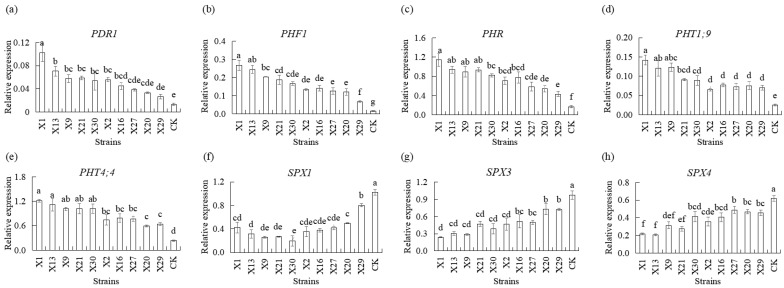
Transcript levels of the genes involved in phosphorus transport of different endophytic PSB in Chinese *Cymbidium.* Relative expressions of (**a**) *PDR1*, (**b**) *PHF1*, (**c**) *PHR*, (**d**) *PHT1;9*, (**e**) *PHT4;4*, (**f**) *SPX1*, (**g**) *SPX3*, and (**h**) *SPX4* in roots were determined 24 h after treatment with different endophytic PSB using qPCR. β-actin (Mol013347) was used as an internal reference gene to normalize the amount of template. Means of three replicates and standard errors are presented. The same letter above the column indicates no significant difference among the data in roots, respectively, at *p* < 0.05.

qPCR analysis showed that Chinese *Cymbidium* plants inoculated with endophytic PSB demonstrated significantly upregulated relative expression of *PDR2*, *PHF1*, *PHR1*, *PHT1;9*, and *PHT4;4* in their roots. *PDR2*, *PHF1*, *PHR1*, *PHT1;9*, and *PHT4;4* transcript levels were induced after the plants were inoculated with PSB. Higher transcript levels of *PDR2*, *PHF1*, *PHR1*, *PHT1;9*, and PHT4;4 were observed in plants inoculated with PSB compared to uninoculated plants (CK) (Figure 5a–e). *SPX1*, *SPX3*, and *SPX4* transcripts were downregulated in all plants inoculated with PSB. Higher transcript levels of *SPX1*, *SPX3*, and *SPX4* were observed in uninoculated plants compared to plants inoculated with PSB (Figure 5f–h). No significant difference in *PHO1*, *PHOS32*, and *THO1* transcript levels was observed between plants that were uninoculated (CK) and those that were inoculated with PSB (Figure S2a,b,d). No significant difference between the PSB strains was observed. PS3 transcript levels in PSB showed no significant difference between X9, X21, X2, X16, X27, and X20 strains, on the one hand, and uninoculated PSB (CK), while higher levels were shown in X1, X13, X30, and 29 strains (Figure S2c).

Results indicated that inoculation with endophytic PSB led to higher levels of *PDR2*, *PHF1*, *PHR1*, *PHT1;9*, *PHT4;4*, *SPX1*, *SPX3*, and *SPX4* transcripts inoculated with endophytic PSB in orchid roots, suggesting their participation in phosphorus metabolism.

### 3.6. Colonization of PSB in Orchid Roots and Rhizosphere Soil

**Figure 6:**
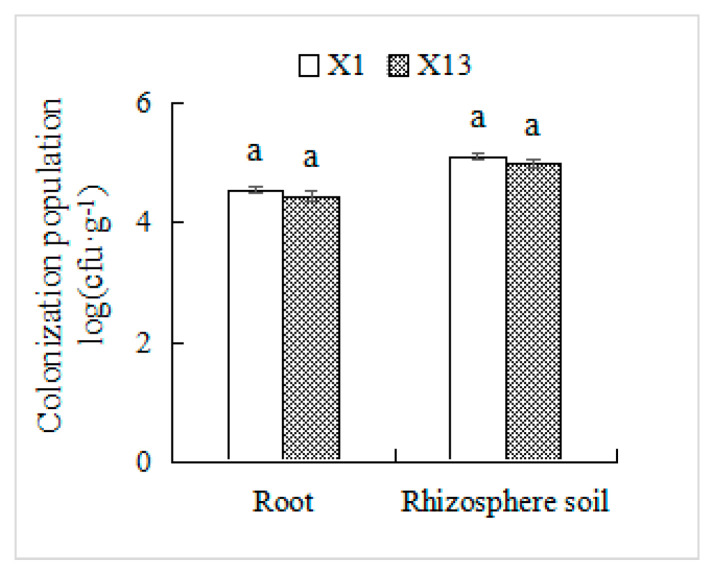
Colonization population of endophytic PSB in Chinese *Cymbidium* root and rhizosphere soil. The root and rhizosphere soil samples of the orchid were diluted and uniformly spread on NA solid medium containing 300 µg·mL^−1^ rifampicin. After incubation at 30 °C for 72 h, the number of colonies on each plate was counted. Means of three replicates and standard errors are presented. Columns with the same letter indicate no significant difference at *p* < 0.05.

To track inoculated strains in plant tissues and soil, we utilized the rifampicin-resistant strains X1 and X13 (selected at 300 μg·mL^−1^) as biological markers. After 30 d of co-cultivation, these antibiotic-resistant strains were successfully re-isolated from both orchid roots and rhizosphere soil using rifampicin-supplemented medium. The colonization densities of X1 reached 2.8 × 10^4^ cfu·g^−1^ in roots and 1.3 × 10^5^ cfu·g^−1^ in rhizosphere soil for X1, with corresponding values of 2.1 × 10^4^ cfu·g^−1^ and 1.0 × 10^5^ cfu·g^−1^ for X13 (Figure 6).

## 4. Discussion

Phosphorus is one of the most critical nutrients for plant growth. Phosphorus deficiency results in reduced plant growth [47]. Although chemical fertilizers can be added to the soil, plants can only utilize low amounts of phosphorus fertilizers [48,49]. As a result, choosing an effective PSB can increase the amount of phosphorus in the plant rhizosphere. In this study, the 30 isolates’ endophytic strains were proven to belong to *Pseudomonas*, *Burkholderia*, *Bacillus*, *Rhizobium*, *Sphingobacterium*, and *Stenotrophomonas* (Table S1). Members of these genera have been previously described as phosphorus solubilizers [8,12,50,51]. Ten endophytic strains showed clear halos on IP and OP solid medium, indicating their ability to dissolve inorganic phosphorus and mineralize organic phosphorus (Table S1). Ten endophytic strains revealed significant differences in phosphate-solubilizing halo zone diameter, colony diameters, and D/d among the strains, suggesting that each strain possesses a distinct capability to solubilize phosphorus (Figure 1 and Figure 2). The D/d produced by the 10 isolates varied from 2.2 to 3.5 mm (Figure 1d and Figure 2e). According to Marra et al. [52], PSB demonstrated a D/d exceeding 2.0, which indicates a general proficiency in phosphate solubilization. The dissolved inorganic phosphorus and mineralized organic phosphorus abilities of the endophytic strains isolated from Chinese *Cymbidium* in this study ranged from 130 to 346 mg·L^−1^ and 7.9 to 151.1 mg·L^−1^, respectively. More importantly, strains X13 and X1 demonstrated a distinctive capacity for simultaneously dissolving inorganic phosphorus and mineralizing organic phosphorus, as evidenced by their superior performance in both liquid and solid media (Figure 1 and Figure 2). This dual functionality is a notable advantage, as many reported PSB strains exhibit proficiency in only one type of phosphorus solubilization [53,54]. The robust and versatile P-solubilizing performance of these orchid-derived endophytic strains, coupled with their innate ability to colonize host plants, provides a strong theoretical basis for their application in the efficient production of orchids and the development of novel microbial fertilizers tailored for phosphorus-limited cultivation systems.

Previous studies have confirmed that distinct mechanisms between inorganic phosphorus and organic phosphorus solubilization by PSB strains are different, and there are also differences in the solubilization of different PSB strains [12]. Previous studies have demonstrated that the phosphorus-solubilizing ability of PSB is primarily attributed to their capacity to produce organic acids and extracellular polysaccharides, which mobilize inorganic phosphorus by reducing soil pH and chelating metal ions [12,17,18,24,55,56]. The negative correlation observed between the levels of soluble phosphorus released by bacteria and the pH of the supernatants in IP liquid medium containing tricalcium phosphate suggests that medium acidification due to organic acid production facilitates bacterial phosphate solubilization. This observation aligns with earlier reports indicating that decomposition of inorganic phosphorus is accompanied by a decrease in pH [18]. Consequently, we measured the content of organic acids and found that inoculation of PSB resulted in production of significant amounts of organic acids, particularly glucuronic acid, lactic acid, citric acid, tartaric acid, and pantothenic acid (Table 1). Numerous studies have demonstrated that one of the mechanisms by which microorganisms solubilize phosphorous and liberate phosphorus from insoluble phosphorous minerals is through production of gluconic acid by periplasmic cell-membrane-bound NADP-dependent glucose dehydrogenase [18,24]. The secreted organic acids lower soil pH, which increases phosphate solubility [17]. Moreover, PSB-produced exopolysaccharides can chelate metal ions, thereby preventing their recombination with phosphate ions [55]. Importantly, these EPS work in coordination with organic acids to effectively solubilize phosphates through combined acidification and chelation effects [56]. Therefore, the higher phosphate-solubilizing ability of strains X13 and X1, as compared to the lower phosphate-solubilizing ability strains X20 and X29, was associated with lower pH levels and higher organic acids and exopolysaccharides concentrations. Furthermore, PSB exhibit a complementary mechanism for enhancing phosphorus availability through the mineralization of organic phosphorus, a process predominantly mediated by extracellular enzymes such as phosphatases. All tested PSB strains demonstrated phosphatase activities, confirming their capacity for enzymatic hydrolysis of organic phosphorus. The higher phosphate-solubilizing ability of strains X13 and X1, when compared to the lower phosphate-solubilizing ability of strains X20 and X29, is associated with higher solubilized organic phosphorus and phosphatase activities (Figure 2). Phosphatases catalyze dephosphorylation of phosphoester bonds in organic compounds, releasing soluble phosphate [57]. This enzymatic system enables PSB to simultaneously mineralize organic phosphorus and solubilize inorganic phosphates, optimizing phosphorus cycling in soil ecosystems.

Given the observed phosphate-solubilizing capabilities of the selected strains in both solid and liquid media, we analyzed their efficacy in solubilizing phosphorus for incorporation into orchids grown in pots under greenhouse conditions. Studying the effects of PSB on the growth of orchids plantlets and unraveling the growth-promoting properties of these bacteria prove to be useful to orchids. Plant height, biomass, and other morphological growth indicators are direct manifestations of the efficiency of plantlets’ growth. As expected, orchids grown in a substrate with tricalcium phosphate and lecithin (CK) as the only source of phosphorus showed clear symptoms of phosphorus deficiency. A significant reduction in leaf length, root length, leaf dry weight, and root dry weight of orchids, as compared to orchids grown from plantlets of different PSB, was observed. Notably, All PSB treatments resulted in significantly greater leaf and root dry weight than the CK treatment (Figure 3). These data showed that most PSB promote the growth of orchid plantlets in terms of plant height and biomass. There were no adverse effects after inoculation of endophytic PSB in orchid plantlets. Soil is the primary reservoir of phosphorus in terrestrial ecosystems, directly influencing the availability of phosphorus for crops [58]. Soil phosphorus content is an important indicator of soil fertility, which reflects the storage and supply of nutrients in the soil. This study revealed that the effects of PSB on soil phosphorus content and microbial metabolites were significantly different (Figure 4). Specifically, the available phosphorus content increased, while total phosphorus decreased significantly (Figure 4). Additionally, the secretion of exopolysaccharides and phosphatase activity of microorganisms in the soil increased significantly, which, in turn, increased the phosphorus content in the orchid leaves and roots (Figure 3 and Figure 4). These results suggest that PSB strains convert insoluble soil phosphorus into a form that is utilizable by plants through the secretion of organic acids, exopolysaccharides, and phosphatases activities. Orchid roots can positively respond to soil phosphorus under phosphorus-deficient conditions so that orchid roots can actively absorb and utilize available phosphorus in soil to promote growth of orchid plantlets.

The uptake and transportation of phosphorus in plants are mediated by *PDR*, *PHF*, *PHO*, *PHT*, and *SPXs*, which play pivotal roles in the regulation of the absorption, utilization, and redistribution of phosphorus by plants [20,21,22]. When plants are subjected to phosphorus deficiency stress, *PHR1* rapidly dissociates from the complex of regulatory genes and negative regulatory factors such as *SPX1* and *SPX2*, thereby regulating the expression of phosphorus starvation response genes [59]. Additionally, regulatory factors involved in plant phosphorus starvation response (PSR) may affect the competition for phosphorus nutrients between plants and microbes, influence the expression levels of PSR transcription factors, and simultaneously stimulate the occurrence of *PSR* in plants [60]. Phosphate transporters also affect the phosphorous solubilization efficiency of the strain. Most bacteria have phosphate transport systems, which are activated when phosphorous levels are limited [61,62]. In this work, we evaluated the effect of PSB on the transcript levels of phosphorus-related genes in orchid root. The relative transcript levels of *PDR2*, *PHF1*, *PHR*, *PHT1;9*, and *PHT4;4* were higher in inoculated orchid roots than in uninoculated roots (Figure 5). These results suggest that these strains’ PSB may solubilize insoluble phosphorus in the soil into soluble phosphorus for plant absorption and utilization, increase the phosphorus content in the environment, and then upregulate the transcript levels of *PDR2*, *PHF1*, *PHR*, *PHT1;9*, and *PHT4;4* in orchid roots. One of the mechanisms by which PSB promotes orchid growth might be the strain-induced release of phosphorus from the soil, providing sufficient phosphorus for the growth of orchids. On the other hand, inoculation of a strain with PSB can upregulate or downregulate the transcript levels of the phosphorus-related genes in orchid roots, thereby enhancing phosphorus uptake from the soil solution and transporting it inside the plant, inside cells, between cells, and between plant organs. These results lay a solid foundation for additional research on the relationships between endophytic PSBs and their host plants. Furthermore, the results of the present work suggested that the endophytic PSBs X1 and X13 can colonize the orchid rhizosphere, as highly efficient phosphate-solubilizing endophytes would probably possess a natural affinity to their host plants This sustained colonization ensures persistent phosphate-solubilizing activity, demonstrating significant potential for promoting growth in Chinese *Cymbidium* orchids.

## 5. Conclusions

This study successfully isolated 10 endophytic bacterial strains with dual phosphate-solubilizing capabilities from the roots of Chinese *Cymbidium*. All strains demonstrated the ability to solubilize IP and mineralize OP through the secretion of organic acids and phosphatases. Among them, strains X1 (*Paraburkholderia* sp. Beta-32) and X13 (*Rhizobium freirei* PRF81) exhibited significantly stronger phosphate-solubilizing capacities compared to other strains, showing higher organic acid production, phosphatase activity, and phosphorus solubilization efficiency. Inoculation with these strains enhanced phosphorus uptake and promoted growth in orchids while upregulating the expression of phosphorus transporter-related genes. The most notable effects were observed in plants treated with X1 and X13. This study provides valuable microbial resources and a theoretical foundation for developing specialized microbial phosphorus fertilizers for orchids. Strains X1 and X13 show strong potential for practical applications, and further efforts should focus on field validation and large-scale application research.

## Figures and Tables

**Table 1:** Comparison of organic acid production of different endophytic PSB isolated from Chinese *Cymbidium* (ug·mL^−1^).

Organic Acid	X1	X13	X9	X21	X30	X2	X16	X27	X20	X29
Lactic acid	9.00±1.80cd	17.25 ± 1.95 a	14.14 ± 2.86 abc	16.76 ± 0.31 ab	10.22 ± 2.42 bcd	7.50 ± 1.84 cd	13.39 ± 3.05 abcd	13.14 ± 2.72 abcd	6.97 ± 1.38 d	11.09 ± 0.44 abcd
Maleic acid	0.02±0.00c	0.03 ± 0.00 c	0.04 ± 0.00 c	0.27 ± 0.06 b	1.95 ± 0.08 a	0.01 ± 0.00 c	0.00 ± 0.00 c	0.02 ± 0.00 c	0.03 ± 0.01 c	0.02 ± 0.00 c
Fumaric acid	0.05±0.01b	0.09 ± 0.02 a	0.03 ± 0.00 bc	0.03 ± 0.01 bcd	0.02 ± 0.00 bcd	0.03 ± 0.01 bcd	0.01 ± 0.00 cd	0.02 ±0.01 cd	0.00 ± 0.00 d	0.00 ± 0.00 d
Succinic acid	2.02 ± 0.23 a	1.42 ± 0.01 b	0.81 ± 0.02 c	0.73 ± 0.09 cd	0.53 ± 0.04 def	0.29 ± 0.01 fg	0.38 ± 0.01 ef	0.53 ±0.02 def	0.62 ± 0.02 cde	0.07 ± 0.01 gh
Nicotinic acid	0.12 ± 0.05 ab	0.05 ± 0.02 bcd	0.05 ± 0.01 bcd	0.01 ± 0.01 cd	0.00 ± 0.00 cd	0.11 ± 0.05 ab	0.16 ± 0.05 a	0.04 ± 0.02 bcd	0.04 ± 0.00 bcd	0.10 ± 0.00 abc
Pyroglutamic acid	0.24 ± 0.04 b	0.12 ± 0.02 c	0.19 ± 0.01 b	0.00 ± 0.00 f	0.01 ± 0.00 ef	0.06 ± 0.01 cdef	0.45 ± 0.06 a	0.08 ± 0.00 cde	0.03 ± 0.00 def	0.10 ± 0.00 cd
Ethylmalonic acid	0.02 ± 0.00 a	0.01 ± 0.01 b	0.01 ± 0.00 b	0.01 ± 0.00 b	0.00 ± 0.00 b	0.01 ± 0.01 b	0.01 ± 0.01 b	0.01 ± 0.00 b	0.01 ± 0.00 b	0.01 ± 0.00 ab
Glutaric acid	0.08 ± 0.04 a	0.03 ± 0.01 bc	0.06 ± 0.00 ab	0.01 ± 0.00 c	0.00 ± 0.00 c	0.03 ± 0.01 bc	0.03 ± 0.00 bc	0.03 ± 0.00 bc	0.03 ± 0.00 bc	0.03 ± 0.00 bc
Malic acid	0.45 ± 0.075 a	0.25 ± 0.04 b	0.13 ± 0.02 c	0.11 ± 0.03 c	0.12 ± 0.02 c	0.02 ± 0.01 d	0.00 ± 0.00 d	0.08 ± 0.01 cd	0.16 ± 0.01 c	0.01 ± 0.00 d
5-HydroXymethyl-2-furoic acid	0.38 ± 0.04 a	0.28 ± 0.00 ab	0.25 ± 0.02 abc	0.19 ± 0.00 bc	0.13 ± 0.08 c	0.16 ± 0.05 bc	0.26 ± 0.01 abc	0.24 ± 0.01 abc	0.17 ± 0.06 bc	0.18 ± 0.04 bc
Tartaric acid	14.52 ± 2.80 ab	7.60 ± 0.19 bcde	9.18 ± 0.11 cd	12.00 ± 1.66 bc	13.83 ± 0.22 b	19.04 ± 2.73 a	4.36 ± 0.04 ef	5.44 ± 0.11 def	3.29 ± 0.25 fg	2.03 ± 0.05 fg
Phenylpyruvic acid	0.24 ± 0.02 c	2.70 ± 0.13 a	0.24 ± 0.08c	0.08 ± 0.03 c	0.04 ± 0.02 c	0.05 ± 0.01 c	2.14 ± 0.18 b	0.15 ± 0.00 c	0.10 ± 0.01 c	0.16 ± 0.03 c
Phenyllactic acid	0.06 ± 0.00 b	2.75 ± 1.09 a	0.09 ± 0.00 b	0.16 ± 0.00 b	0.08 ± 0.04 b	0.04 ± 0.03 b	0.33 ± 0.02 b	0.14 ± 0.05 b	0.07 ± 0.03 b	0.17 ± 0.00 b
Vanillic acid	0.00 ± 0.00 b	0.00 ± 0.00 b	0.00 ± 0.00 b	0.90 ± 0.18 a	0.00 ± 0.00 b	0.00 ± 0.00 b	0.00 ± 0.00 b	0.00 ± 0.00 b	0.00 ± 0.00 b	0.00 ± 0.00b
PyridoXine	0.02 ± 0.01 a	0.00 ± 0.00 b	0.00 ± 0.00 b	0.00 ± 0.00 b	0.00 ± 0.00 b	0.00 ± 0.00 b	0.00 ± 0.00 b	0.00 ± 0.00 b	0.00 ± 0.00 b	0.00 ± 0.00 b
Suberic acid	0.01 ± 0.00 cd	0.02 ± 0.00 cd	0.00 ± 0.00 d	0.00 ± 0.00 d	0.00 ± 0.00 d	0.04 ± 0.00 bc	0.07 ± 0.01 a	0.06 ± 0.03 ab	0.04 ± 0.01 bc	0.00 ± 0.00 d
Hippuric acid	0.00 ± 0.00 d	0.01 ± 0.00 d	0.00 ± 0.00 d	0.00 ± 0.00 d	0.01 ± 0.01 d	0.00 ±0.00 d	0.02 ± 0.00 cd	0.06 ± 0.03 ab	0.07 ± 0.01 a	0.04 ± 0.00 bc
Citric acid	19.37 ± 1.14 a	3.52 ± 0.25 bc	4.21 ± 0.92 b	2.18 ± 0.74 cd	3.94 ± 0.72 bc	1.33 ± 0.07 de	1.32 ± 0.50 de	0.31 ± 0.01 e	3.76 ± 0.09 bc	0.69 ± 0.01 de
D-Glucuronic acid	27.65 ± 3.82 a	23.21 ± 0.33 b	19.21 ± 1.48 c	14.74 ± 0.68 d	14.99 ± 1.60 d	11.60 ± 1.80de	12.61 ± 0.94 de	12.01 ± 0.92 de	9.01 ± 1.84 ef	5.83 ± 0.70 f
Pantothenic acid	1.23 ± 0.35 c	3.75 ± 0.29 a	1.22 ± 0.18 c	0.16 ± 0.04 d	0.62 ± 0.51 cd	0.53 ± 0.08 cd	3.75 ± 0.26 a	1.24 ± 0.63 c	1.22 ± 0.14 c	2.39 ± 0.07 b
Total organic acids	75.40 ± 10.41 a	63.08 ± 4.35 ab	49.83 ± 5.71 bc	48.33 ± 3.85 bc	46.69 ± 5.75 bc	40.84 ± 6.71 cd	39.30 ± 5.15 cd	33.60 ± 4.57 cd	25.63 ± 3.85 d	22.92 ± 1.36 d

Means of three replicates and standard errors are presented, the same letter in each line indicates no significant difference at *p* < 0.05.

## Data Availability

The original contributions presented in the study are included in the article/Supplementary Materials, further inquiries can be directed to the corresponding authors.

## Supplementary Materials

The following supporting information can be downloaded at: https://www.mdpi.com/article/10.3390/microorganisms13102229/s1, Figure S1: Maximum likelihood phylogenetic tree of endophytic bacterial strains isolated from Chinese *Cymbidium* based on 16S rDNA gene sequences.; Figure S2: Transcript levels of the genes involved in phosphorus transport of different endophytic PSB in Chinese *Cymbidium*; Table S1: Preliminary identification of phosphate solubilization ability of bacterial strains isolated from *Cymbidium* orchids; Table S2: Strain names and their NCBI database accession numbers; Table S3: Primer sequences used for RT-qPCR and the accession numbers of the analyzed genes.
