# Supplementary material for: Isolation of Endophytic Phosphate-Solubilizing Bacteria from Chinese Cymbidium (Cymbidium spp.) Orchid Roots
Source: Microorganisms. 2025 Sep 23;13(10):2229. doi: 10.3390/microorganisms13102229 (PMC12566214; doi:10.3390/microorganisms13102229)
Supplement: Supplementary file 1 [file microorganisms-13-02229-s001.zip › microorganisms-3857346-supplementary.pdf]

microorganisms

MDPI

Supporting Information

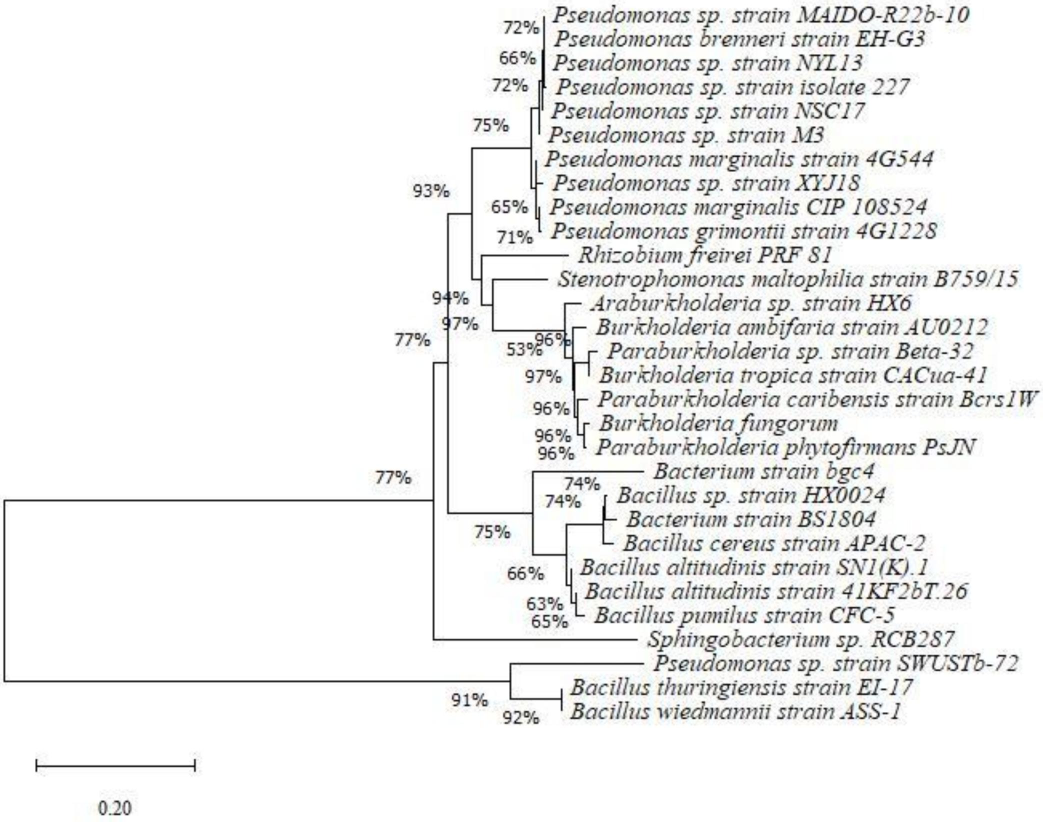

Figure S1. Maximum likelihood phylogenetic tree of endophytic bacterial strains isolated from Chinese *Cymbidium* based on 16S rDNA gene sequences.

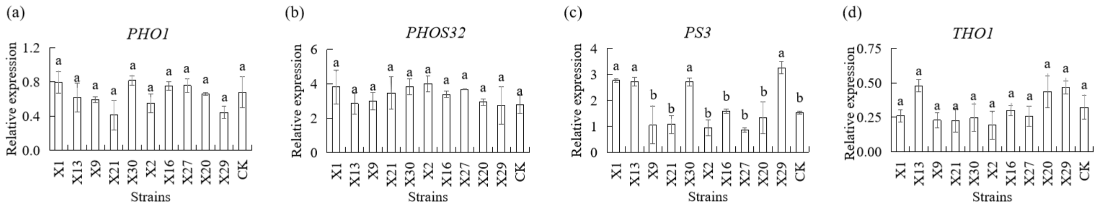

Figure S2. Transcript levels of the genes involved in phosphorus transport of different endophytic PSB in Chinese *Cymbidium*. Relative expressions of (a) *PHO1*, (b) *PHOS32*, (c) *PS3*, and (d) *THO1* in roots were determined 24 h after treatment with different endophytic PSB using qPCR. β-actin (Mol013347) was used as an internal reference gene to normalize the amount of template. Means of three replicates and standard errors are presented. The same letter above the column indicates no significant difference among the data in roots, respectively, at $p<0.05$.

 https://doi.org/10.3390/microorganisms13102229

Table S1. Preliminary identification of phosphate solubilization ability of bacterial strains isolated from *Cymbidium* orchids.

Variety	Strain code	Strains	Homology（%）	Dissolved inorganic phosphorus	Mineralized organic phosphorus
*Hybrid *Cymbidium* ‘Xiao shen tong’	X1	***Paraburkholderia* sp. strain Beta-32**	**99**	+	+
	X2	***Pseudomonas* sp. strain SWUSTb-72**	**99**	+	+
*Hybrid *Cymbidium* ‘Guo qing hong’	X3	*Pseudomonas* sp. strain NYL13	99	-	-
	X4	*Pseudomonas* sp. strain MAIDO-R22b-10	99	-	-
	X5	*Pseudomonas* sp. strain M3	100	-	-
	X6	*Araburkholderia* sp. strain HX6	99	-	-
	X7	*Bacterium* strain bgc4	94	-	-
	X8	*Pseudomonas* sp. strain isolate 227	99	+	-
	X9	***Burkholderia ambifaria* strain AU0212**	**100**	+	+
*Hybrid *Cymbidium* ‘Yu huan fei’	X10	*Paraburkholderia caribensis* strain Bcrs1W	100	-	-
	X11	*Pseudomonas marginalis* CIP 108524	99	-	-
	X12	*Bacillus* sp. strain HX0024	99	+	-
	X13	***Rhizobium freirei* PRF 81**	**100**	+	+
*Hybrid *Cymbidium* ‘S9-A’	X14	*Pseudomonas* sp. strain XYJ18	99	-	-
	X15	*Bacillus altitudinis* strain SN1(K).1	99	-	-
	X16	***Burkholderia fungorum***	**100**	+	+
*Hybrid *Cymbidium* ‘K42’	X17	*Bacillus cereus* strain APAC-2	98	-	-
	X18	*Stenotrophomonas maltophilia* strain B759/15	99	+	-
	X19	*Pseudomonas grimontii* strain 4G1228	99	+	-
	X20	***Pseudomonas brenneri* strain EH-G3**	**100**	+	+
	X21	***Paraburkholderia phytofirmans PsJN***	**100**	+	+
	X22	*Bacterium* strain BS1804	98	+	-
*Hybrid *Cymbidium* ‘K29’	X23	*Pseudomonas marginalis* strain 4G544	99	-	-
	X24	*Bacillus altitudinis* strain 41KF2bT.26	100	-	-
	X25	*Bacillus pumilus strain CFC-5*	99	-	-
	X26	*Pseudomonas* sp. strain NSC17	99	+	-
	X27	**Bacillus thuringiensis strain EI-17**	**99**	+	+
	X28	*Sphingobacterium* sp. RCB287	98	+	-
	X29	***Bacillus wiedmannii* strain ASS-1**	**100**	+	+
	X30	***Burkholderia tropica* strain CACua-41**	**100**	+	+

*parents of hybrids unknown.*parents of hybrids unknown. Bold strains were selected for further investigation.

Table S2 Strain names and their NCBI database accession numbers

Strain code	Accession	Strain names
X1	SAMN48922071	*Paraburkholderia* sp. strain Beta-32
X2	SAMN48922072	*Pseudomonas* sp. strain SWUSTb-72
X9	SAMN48922073	*Burkholderia ambifaria* strain AU0212
X13	SAMN48922074	*Rhizobium freirei* PRF 81
X16	SAMN48922075	*Burkholderia fungorum*
X20	SAMN48922076	*Pseudomonas brenneri* strain EH-G3
X21	SAMN48922077	*Paraburkholderia phytofirmans* PsJN
X27	SAMN48922078	*Bacillus thuringiensis* strain EI-17
X29	SAMN48922079	*Bacillus wiedmannii* strain ASS-1
X30	SAMN48922080	*Burkholderia tropica* strain CACua-41

Table S3. Primer sequences used for RT-qPCR and the accession numbers of the analyzed genes.

Gene name	(5′-3′) Forrward	(5′-3′) Reverse
PHR1	GAGAAGGATAGGGCTGAACAAG	GGAGACTGAGATCCAAGAGAGA
PHT1;9	TTCTTCGGTTGGTTAGGAGATAAG	GAACGACAGTCCAGATCCAATAG
PDR2	CCATGCTGGCATAGCTCTATTA	GATCTTGGCTTCTTCTGCTTTG
PHO1	GCTGCTGTTCGATGGAAGTA	TCCCAGTAGAGCTGGTAGAAA
SPX1	CTTTCTTCACCACTGACCTTCT	TCACCTTGACCGCCATTATC
SPX3	GCTCCACTTATGGTCAGTTCTC	CTATTGAGTTGGGATGGGAGATG
SPX4	CGCAGGCCTTGTAAAGATACT	CGTGAAGAAGGGCTGATGAA
PS3	GGTGCTGGGTACTGGTTAAA	CCAACTACAGCCACAACAGA
THO1	CTTAACCAGCCCGTGAAAGA	GGCAAACCTGGTCGTAAGATA
PHOS32	CGCATCAGAAACTTGAGGAAGA	TGTGGATCTTGAAAGGGATGTG
PHOT2	CTGCCAATCGTTTGGGATCTA	GGAACATCCAGCTCAGGAATAC
PHF1	GTTGGTCCTCAGAAGTGTCTTG	CCTTCAAACTTGGCCACTCTAT
PHT4;4	TGGTGGTATTTGGGCAGATAAG	CTTAGCAGCAATAGGCGTAAGA
β -actin (Mol013347)	ATTGGCTTCTGTCTCGTCTG	CATTACATGGTGAACTAAGGGTA
